# Nonlinear Vibration Study Based on Uncertainty Analysis in MEMS Resonant Accelerometer

**DOI:** 10.3390/s20247207

**Published:** 2020-12-16

**Authors:** Yan Li, Linke Song, Shuai Liang, Yifeng Xiao, Fuling Yang

**Affiliations:** School of Mechanical Electronic & Information Engineering, China University of Mining and Technology-Beijing, Beijing 100083, China; 201572@cumtb.edu.cn (Y.L.); SQT1900402037@student.cumtb.edu.cn (L.S.); SQT2000402038@student.cumtb.edu.cn (S.L.); ZQT2000402056@student.cumtb.edu.cn (Y.X.)

**Keywords:** nonlinear vibration, uncertainty analysis, sample-based stochastic model, experimental verification, MEMS resonant accelerometer

## Abstract

This paper aims to develop a resonant accelerometer for high-sensitivity detection and to investigate the nonlinear vibration of the MEMS resonant accelerometer driven by electrostatic comb fingers. First, a nonlinear vibration model of the resonator with comb fingers in a MEMS resonant accelerometer is established. Then, the nonlinear and nonlinear stiffness coefficients are calculated and analyzed with the Galérkin principle. The linear natural frequency, tracking error, and nonlinear frequency offset are obtained by multi-scale method. Finally, to further analyze the nonlinear vibration, a sample-based stochastic model is established, and the uncertainty analysis method is applied. It is concluded from the results that nonlinear vibration can be reduced by reducing the resonant beam length and increasing the resonant beam width and thickness. In addition, the resonant beam length and thickness have more significant effects, while the resonant beam width and the single concentrated mass of comb fingers have little effect, which are verified by experiments. The results of this research have proved that uncertainty analysis is an effective approach in nonlinear vibration analysis and instructional in practical resonant accelerometer design.

## 1. Introduction

The MEMS (micro-electro-mechanical system) resonant accelerometer can directly convert acceleration into frequency output. It benefits from having small size, light weight, low power, low cost, high resolution, good stability, wide dynamic range and quasi-digital output [1] etc. It is widely used in inertial navigation, seismic detection, intelligent robots and other fields, which has become an important development direction of MEMS sensors. However, when it is vibrating with large amplitude, hard spring nonlinearity of the resonator arises due to the small size of the resonator, which reduces the precision and even causes the sensor to be out of order. Therefore, the research on the nonlinear dynamics of the MEMS resonant accelerometer is of great significance to improve the precision and ensure its normal running.

In recent years, there have been various reports on the nonlinear research of the MEMS resonant accelerometer. In terms of temperature characteristics, Zhang proposed a nonlinear dynamic model of the resonant beam of the differential resonant accelerometer based on Hamilton’s principle under varying temperature conditions, which could completely describe and analyze the nonlinear behavior of the resonant cavity [2]. Defoort et al. used the nonlinear amplitude frequency coupling effect to compensate for the resonator’s passive temperature, which reduced silicon’s temperature coefficient of frequency (TCF) to a level comparable with that of an AT-quartz resonator [3]. Then, Shin et al. also used the nonlinear amplitude frequency effect to improve the bias stability of resonant accelerometer in a large temperature range. In terms of mechanical coupling [4], Gusso proposed and theoretically studied the nonlinear damping mechanism of the transverse vibration of the double-clamped beam resonator [5]. Zou and Seshia optimized the bias voltage of the resonator by using the phase feedback oscillator circuit, and then improved the noise performance of the MEMS resonant accelerometer in the linear and nonlinear state in the wide band [6]. Juillard et al. studied the properties of nonlinearly operated weakly coupled resonators (WCRs) for resonant sensing applications nonlinear operation of the weak coupling resonator [7]. Lu et al. designed the nonlinear digital gain adjustment for rapid establishment of resonance oscillation and linearity improvement of MEMS vibratory gyroscopes [8]. In terms of process materials, Agarwal et al. established and verified the amplitude frequency dependence (A-F) effect model of MEMS resonators and studied the influence and mechanism of nonlinearity on the frequency stability [9]. Mahmoodi et al. undertook a comprehensive analysis and detailed comparative study on the nonlinearity of two types of microcantilever sensors actuated via a piezoelectric ZnO layer, which showed that the nonlinear relation between the stress and strain in some piezoelectric materials had a considerable effect on the sensor [10]. In 2019, Hashemi Kachapi et al. used Gurtin–Murdoch surface/interface theory to analyze the nonlinear vibration and frequency response of double wall piezoelectric nano resonators based on cylindrical nano shells [11]. Behbahani et al. used methods stemming from the ring dynamics [12] to tune the nonlinearity due to manufacturing imperfection in resonators [13,14], and similar frequency tuning method [15,16,17] can be applied to MEMS gyroscopes and accelerometers. These reports mainly used the methods of nonlinear amplitude frequency effect, phase oscillation feedback circuit, material performance optimization and modeling the manufacturing imperfections to reduce the influence of nonlinear vibration of resonators.

As for the uncertainty analysis method, it was first applied to sheetpile cofferdam design in 1987 [18], and then Padmanabhan and Pitchumani used stochastic model to investigate the influence of the uncertainty in the process and the material on the nonisothermal filling process [19]. After model improvement [20], the sample-based stochastic model was used to study the influence of uncertainty on the variability of refractive index, residual stress, maximum tension and defect concentration in the optical fiber stretching process [21,22,23]. At that time, stochastic model had been applied to safety assessments of technological systems [24], thermosetting-matrix composites fabrication [25], proton exchange membrane (PEM) fuel cells [26]. In 2012, Peng et al. developed the sample-based stochastic model to analyze the influence of the uncertainty of parameters on the solid-liquid-vapor phase change of metal particles and identified the laser fluence had dominant effects [27]. Since then, more applications of the stochastic model have been found in the design optimization of resonators [28], thermal damage of living biological tissues by laser irradiation [29], and fluctuation parameters on flow stability [30]. Therefore, the uncertainty method will be a mighty tool to study the nonlinear dynamics of the MEMS resonant accelerometer.

Shi and Fan et al. applied uncertainty method to investigate the effects of different uncertain parameters in electro-thermal excited MEMS resonant sensor, which demonstrated convincingly that the DC excitation voltage had dominant effects [31]. However, it is more complicated to analyze the nonlinear dynamics of the MEMS resonant accelerometer driven by comb.

In this paper, the nonlinear vibration model of resonant beam which is driven by comb fingers in a MEMS resonant accelerometer is established. After being deduced by the Galérkin principle and multi-scale method, the relation between the equivalent stiffness of the resonator and the geometric parameters of the accelerometer is discussed. In the other hand, the influence of pairs of comb fingers on the nonlinear effect of resonator is analyzed. Furthermore, the connection between the natural frequency-tracking error of the accelerometer caused by the nonlinear vibration and the measured acceleration and the geometric parameters of the resonant beam is investigated. Given the uncertainty distribution of geometric parameters and single concentrated mass of the comb fingers due to fabricating errors, the sample-based stochastic model will be applied to investigate influence on vibrating nonlinearity including linear natural frequency, nonlinear frequency offset and their ratio. Finally, we will design a circuit experiment to verify the validity of the uncertainty method.

## 2. Working Principle and Theoretical Analysis

### 2.1. Working Principle

**Figure 1:**
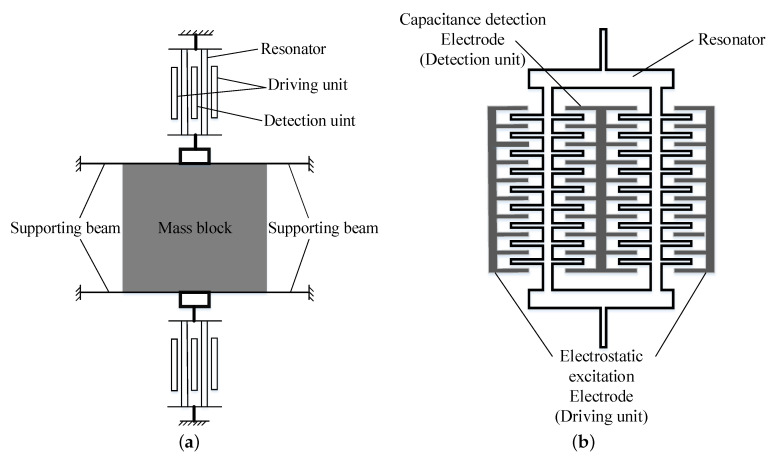
(**a**) structural schematic diagram and (**b**) schematic diagram of drive/detection units.

The MEMS resonant accelerometer mainly includes the mass block, support beams, resonators, drive units and detection units. Each resonator consists of two identical resonant beams. The working principle of the MEMS resonant accelerometer is shown in Figure 1a. The resonator works in the resonant state, and the acceleration acts on the mass block. When the natural frequency of the resonator changes under the inertial force along the axial direction, the drive unit excites the resonator to vibrate and maintains the resonant state. Meanwhile, the detection unit detects the vibration signal, and the drive unit tracks the natural frequency of the resonator controlled by the closed-loop feedback control circuit. It guarantees the frequency of the excitation force is always consistent with the natural frequency of the resonator. Meanwhile, the detection unit detects the vibration signal, and the drive unit tracks the natural frequency of the resonator controlled by the closed-loop feedback control circuit, which guarantees the frequency of the excitation force is always consistent with the natural frequency of the resonator. According to the vibration signal detected by the detection unit, the change of natural frequency can be obtained, and then the measured acceleration can be converted. Figure 1b shows a schematic of the drive/detection units. When the driving voltage (AC and DC bias voltage) is applied to the fixed comb fingers, the alternating electric field will generate a lateral driving force. Under the action of the lateral driving force, the active comb fingers will generate reciprocating vibration relative to the fixed comb fingers. When the frequency of the driving voltage is consistent with the natural frequency of the active comb fingers, the active comb fingers will resonate, and the resonant frequency of the resonator will be obtained through the detection.

### 2.2. Nonlinear Vibration Model of Resonator with Comb Fingers

**Figure 2:**
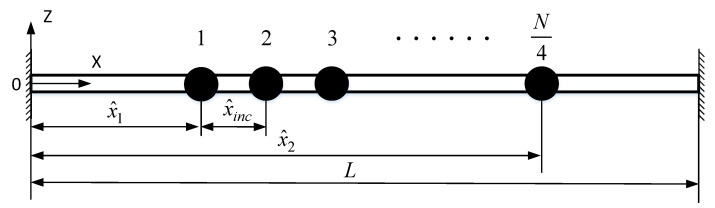
Equivalent mechanical model of resonant beam with comb fingers.

Figure 2 shows the equivalent mechanical model of the resonator single beam in the MEMS resonant accelerometer proposed in Figure 1. Neglecting the moment of inertia of the comb fingers, the comb finger is simplified as a particle attached to the double-clamped resonant beam. Taking the nonlinear geometric effect into account, the equation of motion of the resonant beam is obtained in line with Euler-Bernoulli beam modeling when the electrostatic force is considered. The lateral deflection $\hat{w}\left({\hat{x}}_{i},\hat{t}\right)$ is

(1) $$\begin{array}{cc} \; & \rho BH\frac{\partial^{2}\hat{w}}{\partial {\hat{t}}^{2}}+EI\frac{\partial^{4}\hat{w}}{\partial {\hat{t}}^{4}}+c\frac{\partial \hat{w}}{\partial \hat{t}}-[\frac{EBH}{2L}\int_{0}^{L}(\frac{\partial \hat{w}}{\partial \hat{x}}{)}^{2}d\hat{x}+N_{a}+N_{r}]\frac{\partial^{2}\hat{w}}{\partial {\hat{x}}^{2}}+\sum_{i=1}^{N/4}m_{c}\frac{\partial^{2}\hat{w}\left({\hat{x}}_{i},\hat{t}\right)}{\partial {\hat{t}}^{2}}\delta \left(\hat{x}-{\hat{x}}_{i}\right)\\ \; & =\sum_{i=1}^{N/4}q\left(\hat{t}\right)\delta \left(\hat{x}-{\hat{x}}_{i}\right) \end{array}$$

The boundary conditions of the double-clamped resonant beam are

(2) $$\begin{array}{cc} \frac{\partial \hat{w}\left(0,\hat{t}\right)}{\partial \hat{x}} & =\hat{w}\left(0,\hat{t}\right)=0\\ \frac{\partial \hat{w}\left(L,\hat{t}\right)}{\partial \hat{x}} & =\hat{w}\left(L,\hat{t}\right)=0 \end{array}$$

$q(\hat{t})$ is the electrostatic force generated by a single driving comb finger, which is defined as

(3) $$q\left(\hat{t}\right)=\frac{\varepsilon_{0}b_{e}U_{p}U_{d}}{g}\cos\left(\hat{\omega }\hat{t}\right)$$

where ρ is the material density. L,B and H represent the length, width and thickness of the resonant beam, respectively. *E* is the young’s modulus of the material. *I* is the moment of inertia. $N_{a}$ is the axial load caused by the measured acceleration. $N_{r}$ is the residual stress. $m_{c}$ is the single concentrated mass of the comb finger on the resonant beam. *N* is the pairs of the comb fingers on the resonant beam. ${\hat{x}}_{i}$ is the distance from coordinate of the *i*th concentrated comb mass to the resonant beam end and $\hat{t}$ is time. *c* is the damping coefficient *c* is simplified to be frequency-independent in the neighborhood of resonance and the value is determined by the measured quality factor. δ is the Delta function, which is used to describe the position of the driving moments. $\varepsilon_{0}$ is the permittivity in vacuum. $b_{e}$ is the width of comb finger. *g* is the gap between two plates of comb capacitance. $U_{p}$ and $U_{d}$ are the dc voltage applied to the structure electrode. $\hat{\omega }$ is the working frequency.

Upon Selecting dimensionless parameters

(4) $$w=\frac{\hat{w}}{r}\;x=\frac{\hat{x}}{L}\;t=\hat{t}{\hat{\omega }}_{n}\;\omega =\frac{\hat{\omega }}{{\hat{\omega }}_{n}}$$

where $r=\sqrt{I/BH}$ is the radius of rotation of cross section of resonant beam, the moment of inertia is $I=BH^{3}/12$, and ${\hat{\omega }}_{n}$ is the linear natural frequency of the resonant beam. Equation (1) and the boundary conditions are changed as

(5) $$\begin{array}{cc} \; & \rho BHr{\hat{\omega }}_{n}^{2}\frac{\partial^{2}w}{\partial t^{2}}+\frac{EIr}{L^{4}}\frac{\partial^{4}w}{\partial x^{4}}+cr{\hat{\omega }}_{n}\frac{\partial w}{\partial t}-\frac{r}{L^{2}}[\frac{EBHr^{2}}{2L^{2}}\int_{0}^{1}(\frac{\partial w}{\partial x}{)}^{2}dx+N_{a}+N_{r}]\frac{\partial^{2}w}{\partial t^{2}}+\\ \; & m_{c}r{\hat{\omega }}_{n}^{2}\sum_{i=1}^{N/4}\frac{\partial^{2}w\left(x_{i},t\right)}{\partial t^{2}}\delta \left(\hat{x}-{\hat{x}}_{i}\right)\\ \; & =\sum_{i=1}^{N/4}q\left(t\right)\delta \left(\hat{x}-{\hat{x}}_{i}\right) \end{array}$$

(6) $$\begin{array}{cc} \frac{\partial w(0,t)}{\partial x} & =w(0,t)=0\\ \frac{\partial w(L,t)}{\partial x} & =w(L,t)=0 \end{array}$$

The Galérkin principle is a method to discretize the partial differential equation into a reduced-order model. Assuming the lateral deflection w(x,t) of beam is

(7) $$w\left(x,t\right)=\sum_{i=1}^{n}\phi_{i}\left(x\right)u_{i}\left(t\right)$$

where $\phi_{i}\left(x\right)$ is the ith mode of vibration of the resonant beam, and $u_{i}\left(x\right)$ is the generalized coordinate corresponding to the ith mode [32]. $\phi_{1}\left(x\right)$ is first-order linear undamped mode function of the resonant beam. The solutions of Equations (5) and (6) can be expressed by mode of the resonant function. Through multiplying and integrating from 0 to 1, a reduced-order model in the form of ordinary differential equations can be obtained. Since the distribution of electrostatic force is symmetrical about the midpoint of the resonant beam and the frequency of electrostatic force is close to the first natural frequency, it can be considered that the resonant beam vibrates approximately according to the first mode. According to the undamped free vibration equation of the resonant beam, the first-order vibration mode satisfies the following relationship

(8) $$\frac{EIr}{L^{4}}\phi_{1}^{iv}={\hat{\omega }}_{n}^{2}\rho BHr\phi_{1}+\frac{(N_{a}+N_{r})r}{L^{2}}\phi_{1}^{{}^{\prime \prime }}+m_{c}r{\hat{\omega }}_{n}^{2}\sum_{i=1}^{N/4}\phi_{1}\left({\hat{x}}_{i}\right)\delta \left(\hat{x}-{\hat{x}}_{i}\right)$$

Replacing the first-order vibration mode function and Equation (8) into (5) can obtain

(9) $$\begin{array}{cc} \; & \{\rho BHr{\hat{\omega }}_{n}^{2}\phi_{1}+m_{c}r{\hat{\omega }}_{n}^{2}\sum_{i=1}^{N/4}\phi_{1}\left(x_{i}\right)\delta \left(\hat{x}-{\hat{x}}_{i}\right)\}{\ddot{u}}_{1}+cr{\hat{\omega }}_{n}\phi_{1}{\dot{u}}_{1}+\\ \; & \{\rho BHr{\hat{\omega }}_{n}^{2}\phi_{1}+m_{c}r{\hat{\omega }}_{n}^{2}\sum_{i=1}^{N/4}\phi_{1}\left(x_{i}\right)\delta \left(\hat{x}-{\hat{x}}_{i}\right)\}u_{1}-\frac{EBHr^{3}}{2L^{4}}\phi_{1}^{{}^{\prime \prime }}\int_{0}^{1}{\phi_{1}^{{}^{\prime }}}^{2}dx\cdot u_{1}^{3}\\ \; & =\sum_{i=1}^{N/4}q\left(t\right)\delta \left(\hat{x}-{\hat{x}}_{i}\right) \end{array}$$

Multiplying both sides of Equation (9) by $\phi_{1}\left(x\right)$ and integrating both sides of Equation (9) with respect to *x* from 0 to 1, one obtains

(10) $${\ddot{u}}_{1}+\frac{1}{Q}{\dot{u}}_{1}+k_{1}u_{1}+k_{3}u_{1}^{3}=F_{eq}\cos\left(\omega t\right)$$

where the mechanical quality factor of the resonant beam first-order modal vibration *Q*, the amplitude of equivalent excitation force $F_{eq}$, equivalent linear stiffness coefficient $k_{1}$ and nonlinear stiffness coefficient $k_{3}$ are expressed as

(11) $$\begin{array}{c} \left\{\begin{array}{ccc} \frac{1}{Q} & = & \frac{cr{\hat{\omega }}_{n}\int_{0}^{1}\phi_{1}^{2}dx}{\rho BHr{\hat{\omega }}_{n}^{2}\int_{0}^{1}\phi_{1}^{2}dx+m_{c}r{\hat{\omega }}_{n}^{2}\sum_{i=1}^{N/4}\int_{0}^{1}\phi_{1}\left(x\right)\phi_{1}\left(x_{i}\right)\delta \left(\hat{x}-{\hat{x}}_{i}\right)dx}\\ F_{eq} & = & \frac{\varepsilon_{0}b_{e}U_{p}U_{d}\sum_{i=1}^{N/4}\int_{0}^{1}\phi_{1}\left(x\right)\delta \left(\hat{x}-{\hat{x}}_{i}\right)dx}{\rho BHr{\hat{\omega }}_{n}^{2}g\int_{0}^{1}\phi_{1}^{2}dx+m_{c}r{\hat{\omega }}_{n}^{2}g\sum_{i=1}^{N/4}\int_{0}^{1}\phi_{1}\left(x\right)\phi_{1}\left(x_{i}\right)\delta \left(\hat{x}-{\hat{x}}_{i}\right)dx}\\ k_{1} & = & 1\\ k_{3} & = & -\frac{EBHr^{2}\int_{0}^{1}\phi_{1}^{{}^{\prime \prime }}\phi_{1}dx\int_{0}^{1}{\phi_{1}^{{}^{\prime }}}^{2}dx}{2\rho BHL^{4}{\hat{\omega }}_{n}^{2}\int_{0}^{1}\phi_{1}^{2}dx+2m_{c}L^{4}{\hat{\omega }}_{n}^{2}\sum_{i=1}^{N/4}\phi_{1}^{2}\left(x_{i}\right)} \end{array}\right. \end{array}$$

(12) $$\frac{k_{3}}{k_{1}}=k_{3}=-\frac{EBHr^{2}\int_{0}^{1}\phi_{1}^{{}^{\prime \prime }}\phi_{1}dx\int_{0}^{1}{\phi_{1}^{{}^{\prime }}}^{2}dx}{2\rho BHL^{4}{\hat{\omega }}_{n}^{2}\int_{0}^{1}\phi_{1}^{2}dx+2m_{c}L^{4}{\hat{\omega }}_{n}^{2}\sum_{i=1}^{N/4}\phi_{1}^{2}\left(x_{i}\right)}$$

Equation (12) is the ratio of nonlinear stiffness coefficient to linear stiffness coefficient, which can reflect the nonlinear degree of resonant beam vibration.

**Figure 3:**
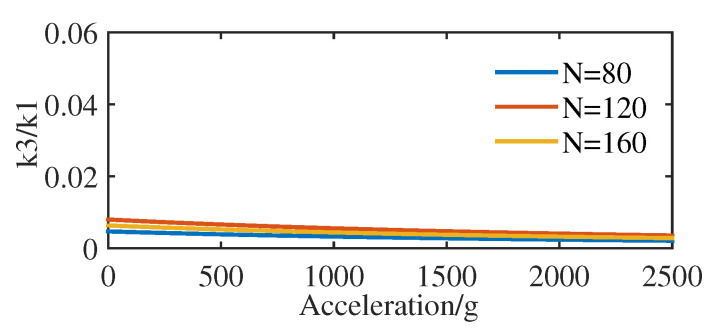
$k_{3}/k_{1}$ curves with the measured acceleration and the pairs of comb fingers.

Supposing that when the accelerometer receives positive acceleration, the resonators receive tensile axial force, and when the accelerometers receive negative acceleration, the resonators receive compressive axial force. According to the determined structural parameters of the resonators with comb fingers, the ratio of nonlinear to linear stiffness coefficient $k_{3}/k_{1}$ with the measured acceleration and the pairs of comb fingers N can be obtained from Equation (12), as shown in the Figure 3. Although increasing the pairs of comb fingers on the resonant beam increases the additional mass of the resonant beam, it can be seen from Figure 3 that increasing the pairs of comb fingers has small effect on the reduction of nonlinearity.

The approximate analytical solution of Equation (10) can be obtained by using the multi-scale method. According to Figure 3 and Equation (12), the nonlinear stiffness coefficient $k_{3}\ll 1$, which can be designated as the small parameter ε in the multi-scale method. Generally, the resonator in resonant accelerometer works in vacuum environment to obtain high mechanical quality factor *Q*, so 1/Q≪1 can be expressed as

(13) $$\begin{array}{cc} \frac{1}{Q} & =2\varepsilon \mu \end{array}$$

(14) $$\begin{array}{cc} \mu & =-\frac{cL^{4}{\hat{\omega }}_{n}\int_{0}^{1}\phi_{1}^{2}dx}{EBHr^{2}\int_{0}^{1}\phi_{1}^{{}^{\prime \prime }}\phi_{1}dx\int_{0}^{1}{\phi_{1}^{{}^{\prime }}}^{2}dx} \end{array}$$

The resonant beam vibrates approximately at their natural frequency when the accelerometer works, so let ω=1+εσ, where σ is the detuning parameter. When the resonant beam vibrates according to the natural frequency, a small excitation amplitude can cause a large vibration of the resonant beam, so let $F_{eq}=\varepsilon K$, where

(15) $$K=-\frac{2\varepsilon_{0}b_{e}U_{p}U_{d}L^{4}\sum_{i=1}^{N/4}\phi_{1}\left(x_{i}\right)}{EBHr^{3}g\int_{0}^{1}\phi_{1}^{{}^{\prime \prime }}\phi_{1}dx\int_{0}^{1}{\phi_{1}^{{}^{\prime }}}^{2}dx}$$

${\hat{\omega }}_{n}$ can be obtained from Equation (12) and considering the relationship between $k_{3}$ and ε

(16) $${\hat{\omega }}_{n}=\sqrt{-\frac{EBHr^{2}\int_{0}^{1}\phi_{1}^{{}^{\prime \prime }}\phi_{1}dx\int_{0}^{1}{\phi_{1}^{{}^{\prime }}}^{2}dx}{2\rho BHL^{4}\varepsilon \int_{0}^{1}\phi_{1}^{2}dx+2m_{c}L^{4}\varepsilon \sum_{i=1}^{N/4}\phi_{1}^{2}\left(x_{i}\right)}}$$

The linear natural frequency $f_{rn}$ is

(17) $$f_{rn}=\frac{{\hat{\omega }}_{n}}{2\pi }$$

So far, Equation (10) can be rewritten as

(18) $${\ddot{u}}_{1}+2\varepsilon \mu {\dot{u}}_{1}+u_{1}+\varepsilon u_{1}^{3}=\varepsilon K\cos\left[\left(1+\varepsilon \sigma \right)t\right]$$

Using the multi-scale method, the amplitude frequency response equation and the phase frequency characteristic equation of the resonant beam are obtained as

(19) $$\hat{\omega }={\hat{\omega }}_{n}+\frac{3{\hat{\alpha }}^{2}}{8r^{2}}{\hat{\omega }}_{n}\varepsilon \pm (\frac{K^{2}r^{2}}{4{\hat{a}}^{2}}-\mu^{2}{)}^{1/2}{\hat{\omega }}_{n}\varepsilon$$

(20) $$\hat{\omega }={\hat{\omega }}_{n}+\frac{3K^{2}{\hat{\omega }}_{n}\varepsilon }{32\mu^{2}}\sin^{2}\gamma -\mu \varepsilon {\hat{\omega }}_{n}\cot\gamma$$

where γ is the phase-shift and $\hat{\alpha }$ is the amplitude of resonant beam.

### 2.3. Natural Frequency-Tracking Error Caused by Nonlinear Vibration

When the phase-locked closed-loop circuit is used to track the natural frequency of the accelerometer, the phase-shift γ of the resonant beam is locked at π/2. Substituting γ=π/2 into Equation (20), the vibration frequency of the resonant beam is expressed as

(21) $$\hat{\omega }={\hat{\omega }}_{n}+\frac{3K^{2}{\hat{\omega }}_{n}\varepsilon }{32\mu^{2}}$$

The frequency-tracking error of the accelerometer can be obtained by Equation (21)

(22) $$E_{r}=\frac{3}{8}Q^{2}K^{2}\varepsilon^{3}{\hat{\omega }}_{n}$$

The nonlinear frequency offset $f_{off}$ is

(23) $$f_{off}=\frac{E_{r}}{2\pi }$$

**Figure 4:**
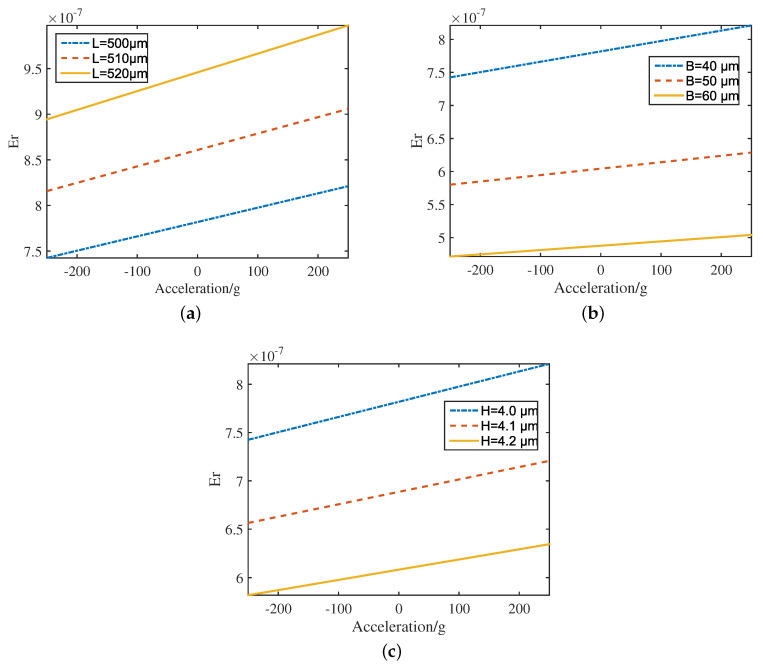
The natural frequency-tracking error with (**a**) length, (**b**) width and (**c**) thickness in accelerometer caused by nonlinear vibration (Q=10,000, $U_{p}=0.50$ V, $U_{d}=0.05$ V).

Figure 4 presents the change curve of the natural frequency-tracking error of the accelerometer caused by the nonlinear vibration with the measured acceleration and the geometric parameters of the resonant beam. As seen from Figure 4, when the measured acceleration increases, the frequency-tracking error increases with the increase of resonant beam length L (Figure 4a) and decreases with the increase of resonant beam width B and thickness H (Figure 4b,c). Therefore, the nonlinear effect can be reduced by decreasing the length and increasing the width and thickness of the resonant beam.

## 3. Uncertainty Analysis Method

### 3.1. Uncertainty Analysis Method

**Figure 5:**
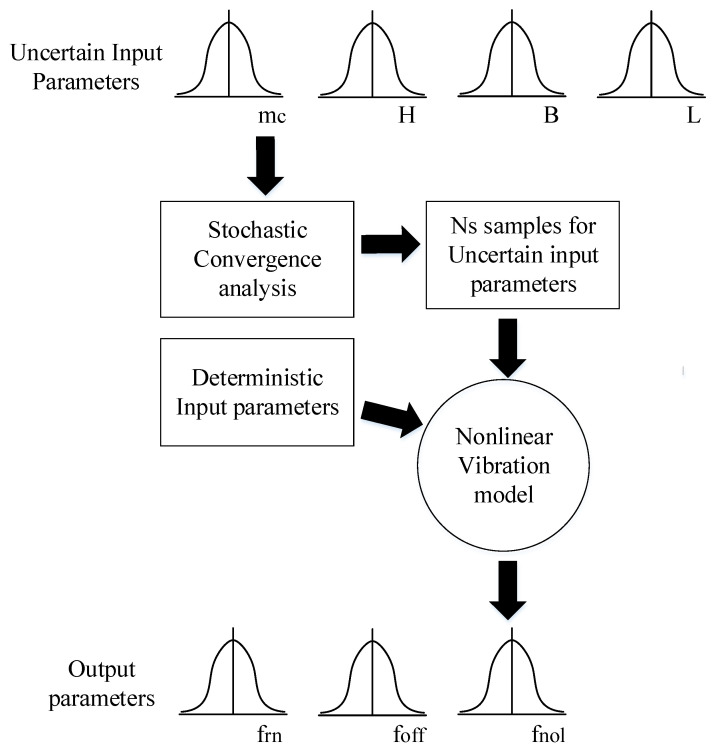
The sample-based stochastic model.

Based on the nonlinear vibration model of resonator, the nonlinear dynamics of MEMS resonant accelerometers is studied by using the sample-based stochastic model. Figure 5 illustrates the detailed procedure on how the sample-based stochastic modeling is realized based on the specific deterministic physical modeling for nonlinear vibration of resonator described with random selected stochastic instances. First, the input parameters are selected, and the degree of change is quantified. Then, through stochastic convergence analysis, a proper number of sample combinations of input parameters are determined, and the uncertainties of input parameters are calculated by the previously established deterministic physical model. Finally, the variability of output parameters is quantified according to the uncertainty of input parameters.

In this study, we focus on the uncertainty in four parameters: the length L, width B, thickness H and single concentrated mass of the comb finger $m_{c}$ of the resonant beam. The uncertainty of all the input parameters is assumed to obey the Gaussian distribution, which is commonly used to represent uncertain parameters of a physical model [20,26]. After determining the distribution of the input parameters, the Monte Carlo sampling method (MCS) is used to randomly select the input parameters from the given Gaussian distribution to obtain a sample combination. In the process of stochastic convergence analysis, with the increase of the number of samples, the mean value and standard deviation of input parameters will converge to the nominal mean value and standard deviation of Gaussian distribution, then the minimum sample value of input parameters will be obtained. To measure the uncertainty of input parameters, the coefficient of variance (COV) defined as σ/μ represents the uncertainty degree of the input parameters, where the average value (μ) is represented by the nominal value of the uncertainty parameters, and the standard deviation (σ) represents the variability of the input parameters.

The intriguing output parameters consist of the linear natural frequency $f_{rn}$, the nonlinear frequency offset $f_{off}$ and the scaling factor $f_{nol}$ defined as $f_{off}/f_{rn}$. Likewise, the number of samples of the output parameters can be obtained by the stochastic convergence analysis. The interquartile range (IQR) defined as the difference between the output parameter values at the 25th percentile (P25) and the 75th percentiles (P75) is used to evaluate the variability of the output parameters distributions [20,26].

### 3.2. Results and Siscussions

According to the finite element analysis method [28], we assume the nominal mean values of L, B, H and $m_{c}$ are 500μm, 40μm, 4μm, $4.66\times 10^{-12}$ kg and the other relevant properties of the resonant beam in this paper are shown in Table 1.

**Figure 6:**
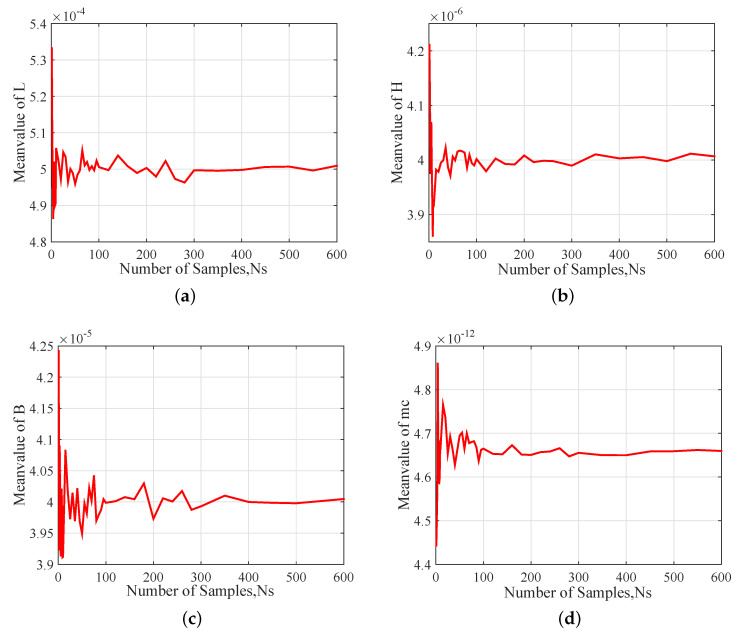
Stochastic convergence analysis of Mean value of input parameters: (**a**) L, (**b**) B, (**c**) H and (**d**) $m_{c}$.

To obtain the number of input samples Ns, the COV of each input parameters is set to be 0.04. Figure 6 shows the results of the stochastic convergence analysis of the mean values of input parameters L, B, H and $m_{c}$. It can be seen that the mean values fluctuate significantly with the decrease of sample numbers but converge as the numbers of samples increases. When $N_{s}=300$, the mean values of the input parameters converge to within 1% for the distributions. In other words, the number of samples of $N_{s}=300$ may be sufficient so far, but it also depends on the standard deviations of input parameters.

**Figure 7:**
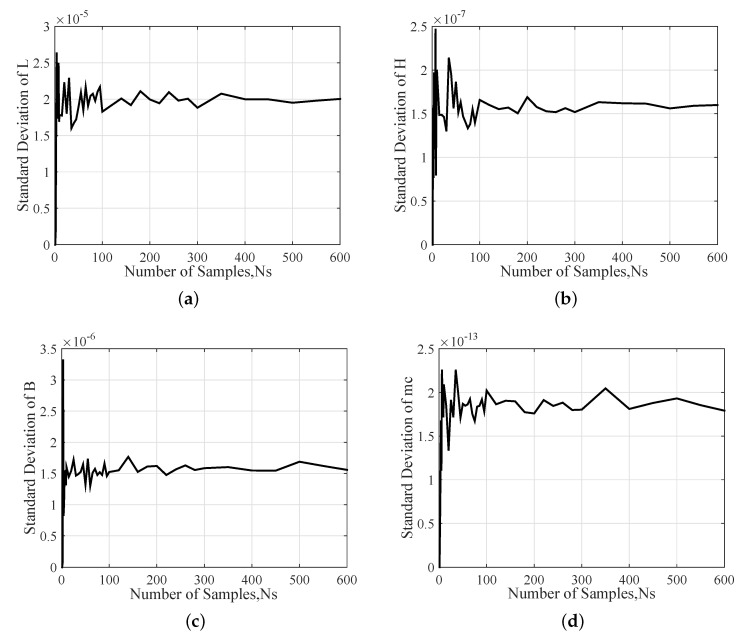
Stochastic convergence analysis of Standard deviation of input parameters: (**a**) L, (**b**) B, (**c**) H and (**d**) $m_{c}$.

The stochastic convergence analysis of the standard deviations of four input parameters are presented in Figure 7, which indicates that there is still a large fluctuation at 300 samples. The standard deviation values converge to within 1.3% for all input parameters while the number of samples exceeding 400.

**Figure 8:**
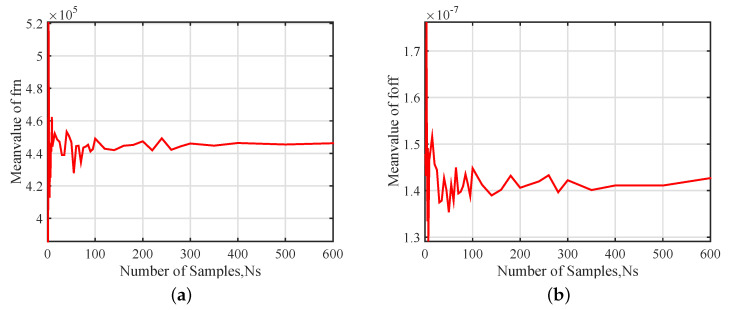

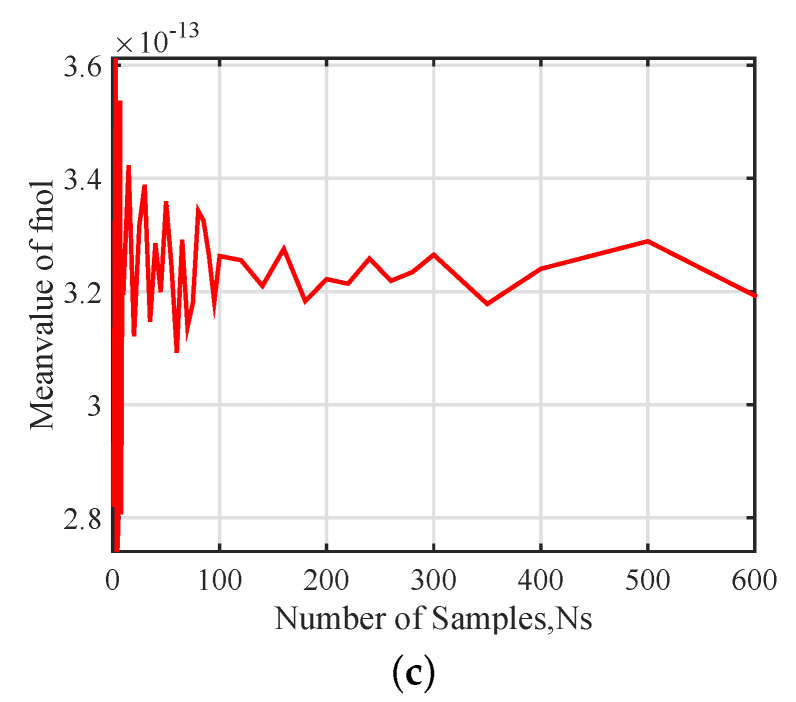
Stochastic convergence analysis of Mean value of output parameters: (**a**) $f_{rn}$, (**b**) $f_{off}$ and (**c**) $f_{nol}$.

Figure 8 presents the stochastic convergence analysis of the mean values of the linear natural frequency $f_{rn}$, the nonlinear frequency offset $f_{off}$ and the scaling factor $f_{nol}$. It can be observed from Figure 8 that the output parameters mean value converges very fast as well. The mean value converges to within 0.034% for $f_{rn}$, 1.11% for $f_{off}$ and 1.48% for $f_{nol}$ when 400 samples are examined.

**Figure 9:**
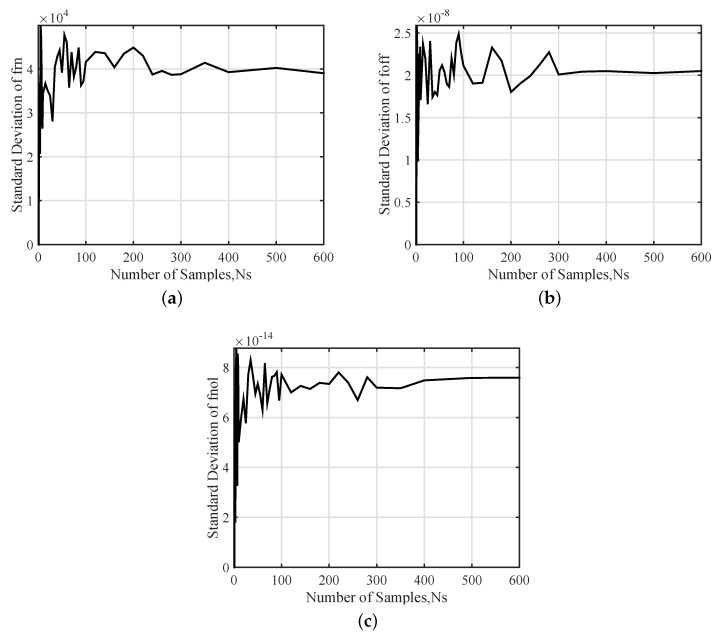
Stochastic convergence analysis of Standard deviation of output parameters: (**a**) $f_{rn}$, (**b**) $f_{off}$ and (**c**) $f_{nol}$.

The results plotted in Figure 9 show the stochastic convergence analysis of the standard deviations of output parameters. The standard deviation converges to within 0.63% for $f_{rn}$, 0.03% for $f_{off}$ and 1.40% for $f_{nol}$ that all output parameters are in 2% when 400 samples are used. In consequence, the minimum number of samples of $N_{s}=400$ is obtained and will be used in the following study.

**Figure 10:**
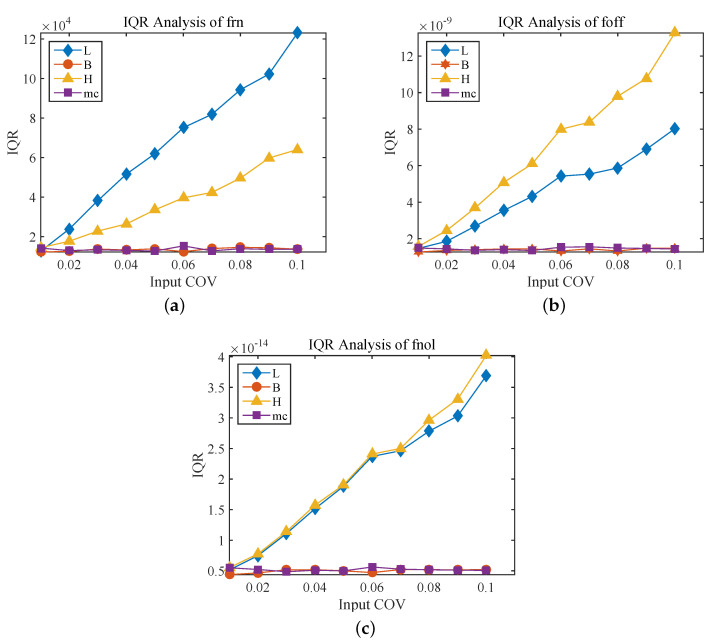
The IQR of output parameters with different COVs of the input parameters. (**a**) IQR Analysis of $f_{rn}$, (**b**) IQR Analysis of $f_{off}$ and (**c**) IQR Analysis of $f_{nol}$.

Figure 10 illustrates the IQRs of the $f_{rn}$, $f_{off}$ and $f_{nol}$ as a function of the COVs of the input parameters L, B, H and $m_{c}$. It should be noted that when the COV of one of the input parameters increases from 0.01 to 0.1, the COVs of other input parameters remain at 0.01. It can be seen from Figure 10a that the IQR of the linear natural frequency $f_{rn}$ significantly increases from about 12.64 kHz to 123.046 kHz with the COV of the length L increases from 0.01 to 0.1. Moreover, when the COV of thickness H increases from 0.01 to 0.1, the IQR of $f_{rn}$ also shows an observable increase from 14.436 kHz to 64.068 kHz. However, the increase of COV of other parameters has very little effect on IQR. The results are consistent with the $f_{rn}$ being a strong function of the length L and the thickness H.

The IQR of the nonlinear natural frequency $f_{off}$ as a function of the COV of various input parameters is presented in Figure 10b, which shows that the IQR of $f_{off}$ increases significantly from $1.562\times 10^{-9}$ Hz to $1.326\times 10^{-8}$ Hz with the COV of the thickness H increasing from 0.01 to 0.1. Meanwhile, when the COV of length L increases from 0.01 to 0.1, the IQR of $f_{off}$ also shows an observable increase from $1.464\times 10^{-9}$ Hz to $8.022\times 10^{-9}$ Hz. It is relatively unaffected by the increase in the COV of other uncertain parameters. Furthermore, the IQR of the scaling factor $f_{nol}$ is a function of the COVs of the input parameters, as depicted in Figure 10c, where it is seen that the COV of the length L and thickness H increasing from 0.01 to 0.1 result in a remarkable increase of the IQR of $f_{nol}$.

The results presented the thickness H and the length L of the resonant beam are the main factors that cause the nonlinear vibration of the MEMS resonant accelerometer, and the influence degree of the thickness H is similar to the length L. This is consistent with the result that the natural frequency-tracking error of the accelerometer caused by the nonlinear vibration changes with the measured acceleration and the geometric parameters of the resonant beam. In addition, the width B of the resonant beam and single concentrated mass of the comb finger $m_{c}$ have little influence on the nonlinearity of the MEMS resonant accelerometer, which is consistent with the pairs of comb fingers making little difference to reduce nonlinearity (Figure 3).

## 4. Experimental Verification

### 4.1. The Equivalent Circuit Model

From the analysis of sample-based stochastic, it can be found that the length L and thickness H of the resonant beam have a great influence on the nonlinear vibration of the resonator. To verify this conclusion, the electromechanical equivalence principle forms a very effective base on which each term of the dynamic Equation (18) is converted into a one-to-one corresponding circuit unit and the equivalent circuit is established. Then, the equivalent circuit of the MEMS resonant accelerometer is established based on the study of its nonlinear vibration theory. In the circuit design, the excitation force only maintains the vibration of the resonant beam and has no influence on the vibration frequency, so the excitation term of Equation (18) is not considered.

**Figure 11:**
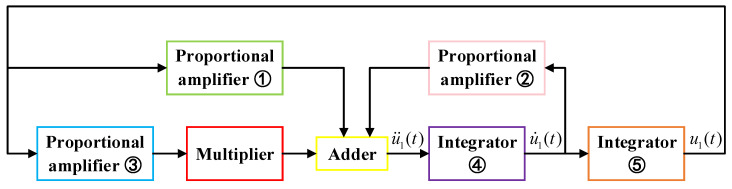
Equivalent circuit model of resonant beam with comb fingers.

The equivalent circuit model of the nonlinear vibration model of resonator with comb fingers is shown in Figure 11. It gives the circuit units corresponding to the terms in the differential Equation (18). $u_{1}\left(t\right)$ is the expression of first-order modal vibration of the resonant beam, which can be obtained by twice integrations of ${\ddot{u}}_{1}\left(t\right)$. The $-u_{1}\left(t\right)$ term can be obtained by using proportional amplifier ① as a feedback loop. The term of nonlinear stiffness coefficient $k_{3}u_{1}^{3}\left(t\right)$ can be obtained by using proportional amplifier ③ and multiplier. The $-u_{1}\left(t\right)$ term corresponds to the proportional amplifier ②. The three terms mentioned above are added by an adder to be the ${\ddot{u}}_{1}\left(t\right)$ term, then the ${\dot{u}}_{1}\left(t\right)$ term and the $u_{1}\left(t\right)$ term can be obtained by integrator ④ and integrator ⑤ respectively. The nonlinear frequency offset is obtained by detecting the difference between the output frequency of $u_{1}\left(t\right)$ and the linear natural frequency calculated by theory.

**Figure 12:**
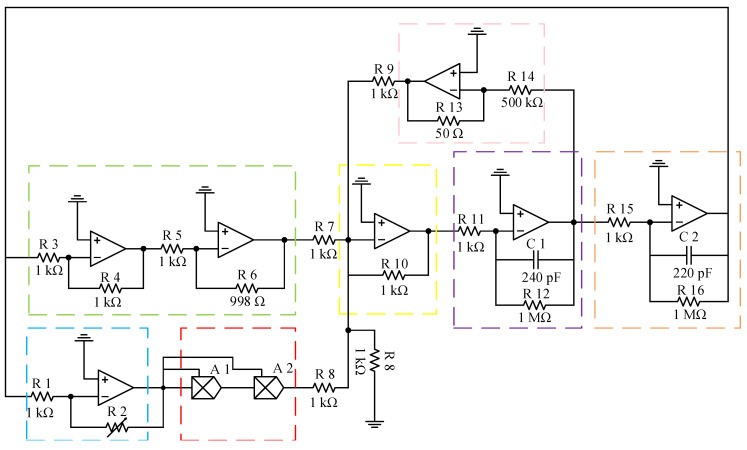
Circuit principle diagram of resonant beam with comb fingers.

The circuit principle diagram of the nonlinear vibration model of resonator with comb fingers is demonstrated in Figure 12. The green box corresponds to the Proportional amplifier ①, the pink box to the Proportional amplifier ②, the yellow box to the Adder, the purple box to the Integrator ④, the orange box to the Integrator ⑤, the blue box to the Proportional amplifier ③, and the red box to the Multiplier. The nonlinear coefficients corresponding to different geometric parameters are obtained by adjusting the feedback resistance R2 of the proportional amplifier in the blue box.

**Figure 13:**
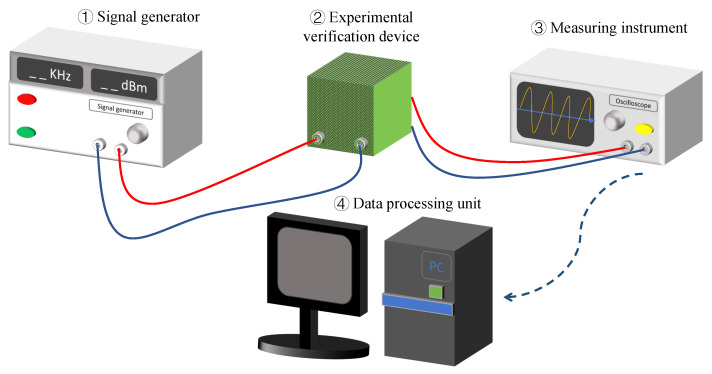
Diagram of experimental setup.

Figure 13 is the diagram of experimental setup. The green box represents the experimental verification module. First, we assume the nominal mean values of L, B, H and $m_{c}$ are 500μm, 40μm, 4μm and $4.66\times 10^{-12}$ kg. Twelve points are sampled for each group of geometric parameters, and to ensure the comparability of the four group of data, one-fortieth of the nominal value of each change is taken. It should be noted that when studying the influence of one geometric parameter, keep the other three geometric parameters as nominal values. As mentioned above, the corresponding nonlinear coefficient $k_{3}$ will also change when the other three parameters are kept unchanged and only one parameter is changed. The change in $k_{3}$ can be achieved by adjusting the variable resistor shown in Figure 12. Secondly, the signal generator gives a signal to the experimental verification device to make it working in a resonant state. Then, adjusting R2 according to the change of geometric parameters, and the measuring instrument records the different frequencies of the output signal. Finally, through the data processing unit analyzes and processes the output frequency recorded by the measuring instrument, the variation of the detected frequency with geometric parameters is obtained. At the same time, the nonlinear frequency offset varying with geometric parameters can be obtained by comparing the detection frequency with the theoretical frequency.

### 4.2. Experimental Results

**Figure 14:**
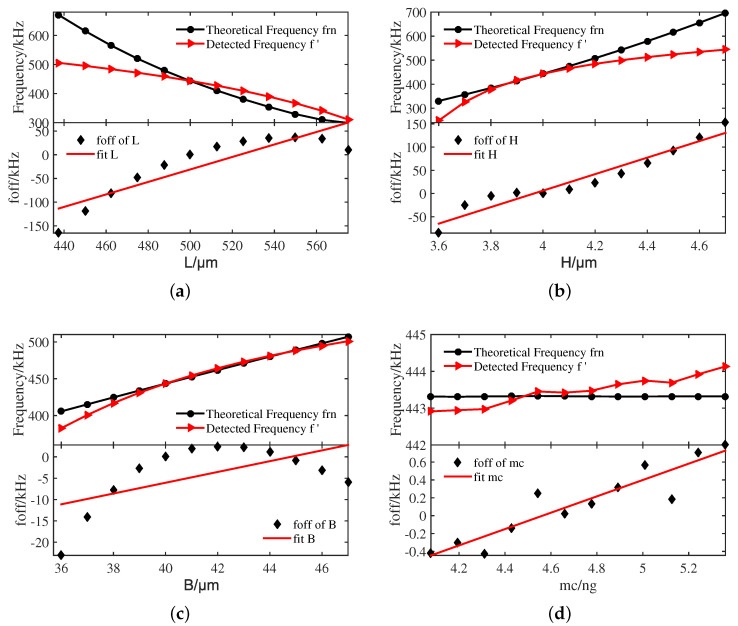
The experimental and theoretical results. (**a**) L, (**b**) H, (**c**) B and (**d**) $m_{c}$.

Figure 14 illustrates the theoretical frequency and detected frequency varying with geometric parameters and the trend fitting curves of the nonlinear frequency offset $f_{off}$. It can be seen from Figure 14a that as the length L increases from 437.5μm to 575μm, both the theoretical linear natural frequency and the nonlinear natural frequency of the circuit output show a decrease trend between 300 kHz and 700 kHz. Moreover, when the length L increases from 437.5μm to 575μm, the nonlinear frequency offset $f_{off}$ shows an observable increase from −150 kHz to 60 kHz in the fitting graph.

Figure 14b presents the theoretical frequency and detected frequency varying with the thickness H. For the convenience of observing the trend, the absolute value of the nonlinear frequency offset point after the nominal value of the resonator thickness H is taken. It can be observed that the theoretical frequency and the detected frequency of the circuit output show an increase trend from 150 kHz to 700 kHz with the thickness H increasing from 3.6μm to 4.7μm. Furthermore, the nonlinear frequency offset $f_{off}$ shows a remarkable increase from −85.156 kHz to 152.802 kHz in the fitting graph, which indicates that the nonlinear frequency offset is also strongly affected by the thickness H.

The results plotted in Figure 14c show the theoretical frequency and detected frequency varying with the width B. It can be seen that as the width B increases from 36μm to 47μm, both the theoretical frequency and the detected frequency of the circuit output show a slightly upward trend from 380 kHz to 510 kHz. Meanwhile, the nonlinear frequency offset $f_{off}$ of the width B increases more tardily from −32 kHz to 5 kHz in the fitting graph than the length L and the thickness H.

Moreover, when the single concentrated mass of the comb finger $m_{c}$ increases from 4.0775 ng to 5.359 ng, as depicted in Figure 14d, it can be seen that neither the theoretical frequency nor the detected frequency of the circuit output changes significantly. The nonlinear frequency offset $f_{off}$ of the single comb finger mass $m_{c}$ varies from −0.416 kHz to 0.7912 kHz, which indicates that the single comb finger mass $m_{c}$ has little effect on the nonlinear frequency offset $f_{off}$.

In conclusion, the detected frequency of the circuit output and the theoretical frequency change significantly with the increase of the length L and the thickness H, followed by B and $m_{c}$. Meanwhile, the length L and the thickness H are the main factor affecting the nonlinear frequency offset $f_{off}$, which is consistent with the results of the uncertainty analysis method.

## 5. Conclusions

- The nonlinear vibration model of resonator with comb fingers has been established. The nonlinear stiffness coefficient $k_{3}$ and the linear stiffness coefficient $k_{1}$ have been calculated and analyzed with the Galérkin principle. The linear natural frequency $f_{rn}$, the nonlinear frequency offset $f_{off}$ and the scaling factor $f_{nol}$ are obtained by multi-scale method.
- According to theoretical analysis, it is found that the pairs of comb fingers have little effect on the nonlinear vibration of the resonator. After further analyzing the relationship between the natural frequency-tracking error and the geometric parameters of the resonant beam, we can find that the frequency-tracking error increases with the increase of the length L of the resonant beam, and decreases with the increase of the width B and thickness H of the resonant beam. The nonlinear effect can be reduced by reducing the length L and increasing the width B and thickness H of the resonant beam.
- Based on the nonlinear vibration model of the resonator and considering uncertainty distributions of structure size due to fabricating errors, a sample-based stochastic model is established to further investigate the effect of input parameters (L, B, H, $m_{c}$) on the output parameters ($f_{rn}$, $f_{off}$, $f_{nol}$). The results show that the length L and thickness H of the resonant beam have a greater influence than the width B and the single concentrated mass of the comb finger $m_{c}$ on the nonlinear vibration of the resonator, which are consistent with the results of theoretical analysis.
- In the experimental verification, the detected frequency of the circuit output and the theoretical frequency change significantly with the increase of the length L and the thickness H, followed by B and $m_{c}$. Meanwhile, the length L and the thickness H are the main factors affecting the nonlinear frequency offset $f_{off}$, which is consistent with the results of the uncertainty analysis method. According to the above conclusions, to reduce the nonlinear characteristics of the sensor, four geometric parameters can be properly adjusted to increase the stiffness and obtain better structural performance.

## Figures and Tables

**Table 1:** Properties of the resonant beam.

Parameter	Expression	Dimensions
Length of the resonant beam	L	500μm
Width of the resonant beam	B	40μm
Thickness of the resonant beam	H	4μm
Pairs of the comb fingers	N	80
Single concentrated mass of comb finger	$m_{c}$	$4.66\times 10^{-12}$ kg
Material density	ρ	$2329\;\mathrm{kg}\cdot m^{-3}$
Young’s modulus of the material	E	$133\;{\mathrm{GP}}_{a}$
Poisson’s ratio	ν	0.278
